# Demonstrating multi-round subsystem quantum error correction using matching and maximum likelihood decoders

**DOI:** 10.1038/s41467-023-38247-5

**Published:** 2023-05-18

**Authors:** Neereja Sundaresan, Theodore J. Yoder, Youngseok Kim, Muyuan Li, Edward H. Chen, Grace Harper, Ted Thorbeck, Andrew W. Cross, Antonio D. Córcoles, Maika Takita

**Affiliations:** 1 IBM Quantum, IBM T.J. Watson Research Center, Yorktown Heights, NY 10598 USA; 2 IBM Quantum, IBM Almaden Research Center, San Jose, CA 95120 USA

**Keywords:** Quantum information, Qubits

## Abstract

Quantum error correction offers a promising path for performing high fidelity quantum computations. Although fully fault-tolerant executions of algorithms remain unrealized, recent improvements in control electronics and quantum hardware enable increasingly advanced demonstrations of the necessary operations for error correction. Here, we perform quantum error correction on superconducting qubits connected in a heavy-hexagon lattice. We encode a logical qubit with distance three and perform several rounds of fault-tolerant syndrome measurements that allow for the correction of any single fault in the circuitry. Using real-time feedback, we reset syndrome and flag qubits conditionally after each syndrome extraction cycle. We report decoder dependent logical error, with average logical error per syndrome measurement in Z(X)-basis of ~0.040 (~0.088) and ~0.037 (~0.087) for matching and maximum likelihood decoders, respectively, on leakage post-selected data.

## Abstract (web summary)

Quantum error correction will be the key to allow large-scale quantum computing operations in the future. Here, the authors use a superconducting qubit system to demonstrate quantum error correction of a distance-three logical qubit in the heavy-hexagon subsystem code.

## Introduction

The outcomes of quantum computations can be faulty, in practice, due to noise in the hardware. To eliminate the resulting faults, quantum error correction (QEC) codes can be used to encode the quantum information into protected, logical degrees of freedom, and then by correcting the faults faster than they accumulate enable fault-tolerant (FT) computations. A complete execution of QEC will likely require: preparation of logical states; realization of a universal set of logical gates, which may require the preparation of magic states; repeated measurements of syndromes; and the decoding of the syndromes for correcting errors. If successful, the resulting logical error rates should be less than the underlying physical error rates, and decrease with increasing code distances down to negligible values.

Choosing a QEC code requires consideration of the underlying hardware and its noise properties. For a heavy-hexagon lattice^1,2^ of qubits, subsystem QEC codes^3^ are attractive because they are well-suited for qubits with reduced connectivities. Other codes have shown promise due to their relatively high threshold for FT^4^ or large number of transversal logical gates^5^. Although their space and time overhead may pose a significant hurdle for scalability, there exist encouraging approaches to reduce the most expensive resources by exploiting some form of error mitigation^6^.

In the decoding process, successful correction depends not only on the performance of the quantum hardware, but also on the implementation of the control electronics used for acquiring and processing the classical information obtained from syndrome measurements. In our case, initializing both syndrome and flag qubits via real-time feedback between measurement cycles can help mitigate errors. At the decoding level, whereas some protocols exist to perform QEC asynchronously within a FT formalism^7,8^, the rate at which the error syndromes are received should be commensurate with their classical processing time to avoid an increasing backlog of syndrome data. Also, some protocols, like using a magic state for a logical *T*-gate^9^, require the application of real-time feed-forward.

Thus, the long term vision of QEC does not gravitate around a single ultimate goal but should be seen as a continuum of deeply interrelated tasks. The experimental path in the development of this technology will comprise the demonstration of these tasks in isolation first and their progressive combination later, always while continuously improving their associated metrics. Some of this progress is reflected in numerous recent advances on quantum systems across different physical platforms, which have demonstrated or approximated several aspects of the desiderata for FT quantum computing. In particular, FT logical state preparation has been demonstrated on ions^10^, nuclear spins in diamond^11^ and superconducting qubits^12^. Repeated cycles of syndrome extraction have been shown in superconducting qubits in small error detecting codes^13,14^, including partial error correction^15^ as well as a universal (albeit not FT) set of single-qubit gates^16^. A FT demonstration of a universal gate set on two logical qubits has recently been reported in ions^17^. In the realm of error correction, there have been recent realizations of the distance-3 surface code on superconducting qubits with decoding^18^ and post-selection^19^, as well as a FT implementation of a dynamically protected quantum memory using the color code^20^ and the FT state preparation, operation, and measurement, including its stabilizers, of a logical state in the Bacon-Shor code in ions^20,21^.

Here we combine the capability of real-time feedback on a superconducting qubit system with a maximum likelihood decoding protocol hitherto unexplored experimentally in order to improve the survivability of logical states. We demonstrate these tools as part of the FT operation of a subsystem code^22^, the heavy-hexagon code^1^, on a superconducting quantum processor. Essential to making our implementation of this code fault-tolerant are flag qubits that, when found to be non-zero, alert the decoder to circuit errors. By conditionally resetting flag and syndrome qubits after each syndrome measurement cycle, we protect our system against errors arising from the noise asymmetry inherent to energy relaxation. We further exploit recently described decoding strategies^15^ and extend the decoding ideas to include maximum likelihood concepts^4,23,24^.

## Results

### The heavy-hexagon code and multi-round circuits

**Fig. 1:**
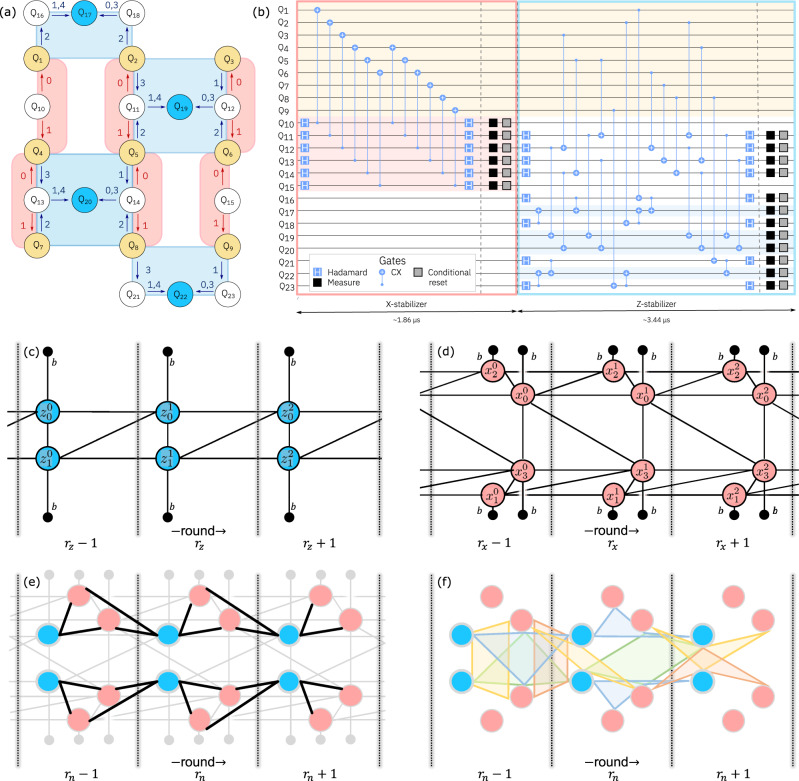
Heavy-hexagon code. **a** *Z* (blue) and *X* (red) gauge operators (eqs. (1) and (2)) mapped onto the 23 qubits required with the distance-3 heavy-hexagon code. Code qubits (*Q*_1_ − *Q*_9_) are shown in yellow, syndrome qubits (*Q*_17_, *Q*_19_, *Q*_20_, *Q*_22_) used for *Z* stabilizers in blue, and flag qubits and syndromes used in *X* stabilizers in white. The order and direction that CX gates are applied within each sub-section (0 to 4) are denoted by the numbered arrows. **b** Circuit diagram of one syndrome measurement round, including both *X* and *Z* stabilizers. The circuit diagram illustrates permitted parallelization of gate operations: those within the bounds set by scheduling barriers (vertical dashed gray lines). As each two-qubit gate duration differs, the final gate scheduling is determined with a standard as-late-as-possible circuit transpilation pass; after which dynamical decoupling is added to data qubits where time permits. Measurement and reset operations are isolated from other gate operations by barriers to allow for uniform dynamical decoupling to be added to idling data qubits. Decoding graphs for three rounds of (**c**) *Z* and (**d**) *X* stabilizer measurements with circuit-level noise allow correction of *X* and *Z* errors, respectively. The blue and red nodes in the graphs correspond to difference syndromes, while the black nodes are the boundary. Edges encode various ways errors can occur in the circuit as described in the text. Nodes are labeled by the type of stabilizer measurement (*Z* or *X*), along with a subscripts indexing the stabilizer, and superscripts denoting the round. **e** Black edges, arising from Pauli *Y* errors on code qubits (and so are just size-2), connect the two graphs in **c** and **d**, but are not used in the matching decoder. **f** The size-4 hyperedges, which are not used by matching, but are used in the maximum likelihood decoder. Colors are just for clarity. Translating each in time by one round also gives a valid hyperedge (with some variation at the time boundaries). Also not shown are any of the size-3 hyperedges.

The heavy-hexagon code we consider is an *n* = 9 qubit code encoding *k* = 1 logical qubit with distance *d* = 3^1^. The *Z* and *X* gauge (see Fig. 1a) and stabilizer groups are generated by

1 $${{{{{{{{\mathcal{G}}}}}}}}}_{Z}=\langle {Z}_{1}{Z}_{2},\,{Z}_{2}{Z}_{3}{Z}_{5}{Z}_{6},\,{Z}_{4}{Z}_{5}{Z}_{7}{Z}_{8},\,{Z}_{8}{Z}_{9}\rangle$$

2 $${{{{{{{{\mathcal{G}}}}}}}}}_{X}=\langle {X}_{1}{X}_{4},\,{X}_{2}{X}_{5},\,{X}_{3}{X}_{6},\,{X}_{4}{X}_{7},\,{X}_{5}{X}_{8},\,{X}_{6}{X}_{9}\rangle$$

3 $${{{{{{{{\mathcal{S}}}}}}}}}_{Z}=\langle {Z}_{1}{Z}_{2}{Z}_{4}{Z}_{5}{Z}_{7}{Z}_{8},\,{Z}_{2}{Z}_{3}{Z}_{5}{Z}_{6}{Z}_{8}{Z}_{9}\rangle$$

4 $${{{{{{{{\mathcal{S}}}}}}}}}_{X}=\langle {X}_{1}{X}_{2}{X}_{4}{X}_{5},\,{X}_{3}{X}_{6},\,{X}_{4}{X}_{7},\,{X}_{5}{X}_{6}{X}_{8}{X}_{9}\rangle$$

The stabilizer groups ${{{{{{{{\mathcal{S}}}}}}}}}_{Z},{{{{{{{{\mathcal{S}}}}}}}}}_{X}$ are the centers of the respective gauge groups ${{{{{{{{\mathcal{G}}}}}}}}}_{Z},{{{{{{{{\mathcal{G}}}}}}}}}_{X}$. This means the stabilizers, as products of gauge operators, can be deduced from measurements of only the gauge operators. Logical operators can be chosen to be *X*_*L*_ = *X*_1_*X*_2_*X*_3_ and *Z*_*L*_ = *Z*_1_*Z*_3_*Z*_7_.

Here we focus on a particular FT circuit, many of our techniques can be used more generally with different codes and circuits. Two sub-circuits, shown in Fig. 1b, are constructed to measure the *X*- and *Z*-gauge operators. The *Z*-gauge measurement circuit also acquires useful information by measuring flag qubits.

We prepare code states in the logical ${\left|0\right\rangle }_{L}$ (${\left|\!+\!\right\rangle }_{L}$) state by first preparing nine qubits in the ${\left|0\right\rangle }^{\otimes 9}$ (${\left|\!+\!\right\rangle }^{\otimes 9}$) state and measuring the *X*-gauge (*Z*-gauge). We then perform *r* rounds of syndrome measurement, where a round consists of a *Z*-gauge measurement followed by an *X*-gauge measurement (respectively, *X*-gauge followed by *Z*-gauge). Finally, we read out all nine code qubits in the *Z* (*X*) basis. We perform the same experiments for initial logical states ${\left|1\right\rangle }_{L}$ and ${\left|-\right\rangle }_{L}$ as well, by simply initializing the nine qubits in ${\left|1\right\rangle }^{\otimes 9}$ and ${\left|-\right\rangle }^{\otimes 9}$ instead.

### Decoding algorithms

In the setting of FT quantum computing, a decoder is an algorithm that takes as input syndrome measurements from an error correcting code and outputs a correction to the qubits or measurement data. In this section we describe two decoding algorithms: perfect matching decoding and maximum likelihood decoding.

The decoding hypergraph^15^ is a concise description of the information gathered by a FT circuit and made available to a decoding algorithm. It consists of a set of vertices, or error-sensitive events, *V*, and a set of hyperedges *E*, which encode the correlations between events caused by errors in the circuit. Figure 1c–f depicts parts of the decoding hypergraph for our experiment.

Constructing a decoding hypergraph for stabilizer circuits with Pauli noise can be done using standard Gottesman-Knill simulations^25^ or similar Pauli tracing techniques^26^. First, an error-sensitive event is created for each measurement that is deterministic in the error-free circuit. A deterministic measurement *M* is any measurement whose outcome *m* ∈ {0, 1} can be predicted by adding modulo two the measurement outcomes from a set ${{{{{{{{\mathcal{A}}}}}}}}}_{M}$ of earlier measurements. That is, for an error-free circuit, $m{=\bigoplus }_{\mu \in {{{{{{{{\mathcal{A}}}}}}}}}_{M}}\mu :={F}_{M}$, where the set ${{{{{{{{\mathcal{A}}}}}}}}}_{M}$ can be found by simulation of the circuit. Set the value of the error-sensitive event to *m* − *F*_*M*_(mod2), which is zero (also called trivial) in the absence of errors. Thus, observing a non-zero (also called non-trivial) error-sensitive event implies the circuit suffered at least one error. In our circuits, error-sensitive events are either flag qubit measurements or the difference of subsequent measurements of the same stabilizer (also sometimes called difference syndromes).

Next, hyperedges are added by considering circuit faults. Our model contains a fault probability *p*_*C*_ for each of several circuit components

5 $$C\in \{{{{{{{{\rm{cx}}}}}}}},\,{{{{{{{\rm{h}}}}}}}},\,{{{{{{{\rm{id}}}}}}}},\,{{{{{{{\rm{idm}}}}}}}},\,{{{{{{{\rm{x}}}}}}}},\,{{{{{{{\rm{y}}}}}}}},\,{{{{{{{\rm{z}}}}}}}},\,{{{{{{{\rm{measure}}}}}}}},\,{{{{{{{\rm{initialize}}}}}}}},\,{{{{{{{\rm{reset}}}}}}}}\}.$$

Here we distinguish the identity operation id on qubits during a time when other qubits are undergoing unitary gates, from the identity operation idm on qubits when others are undergoing measurement and reset. We reset qubits after they are measured, while we initialize qubits that have not been used in the experiment yet. Finally cx is the controlled-not gate, h is the Hadamard gate, and x, y, z are Pauli gates. (see Methods “IBM_Peekskill and experimental details” for more detail). Numerical values for *p*_*C*_ are listed in Methods “IBM_Peekskill and experimental details”.

Our error model is circuit depolarizing noise. For initialization and reset errors, a Pauli *X* is applied with the respective probabilities *p*_init_ and *p*_reset_ after the ideal state preparation. For measurement errors, Pauli *X* is applied with probability ${p}_{{{{{{{{\rm{measure}}}}}}}}}$ before the ideal measurement. A one-qubit unitary gate (two-qubit gate) *C* suffers with probability *p*_*C*_ one of the three (fifteen) non-identity one-qubit (two-qubit) Pauli errors following the ideal gate. There is an equal chance of any of the three (fifteen) Pauli errors occurring.

When a single fault occurs in the circuit, it causes some subset of error-sensitive events to be non-trivial. This set of error-sensitive events becomes a hyperedge. The set of all hyperedges is *E*. Two different faults may lead to the same hyperedge, so each hyperedge may be viewed as representing a set of faults, each of which individually causes the events in the hyperedge to be non-trivial. Associated with each hyperedge is a probability, which, at first order, is the sum of the probabilities of faults in the set.

A fault may also lead to an error which, propagated to the end of the circuit, anti-commutes with one or more of the code’s logical operators, necessitating a logical correction. We assume for generality that the code has *k* logical qubits and a basis of 2*k* logical operators, but note *k* = 1 for the heavy-hexagon code used in the experiment. We can keep track of which logical operators anti-commute with the error using a vector from ${{\mathbb{Z}}}_{2}^{2k}$. Thus, each hyperedge *h* is also labeled by one of these vectors ${\gamma }_{h}\in {{\mathbb{Z}}}_{2}^{2k}$, called a logical label. Note that if the code has distance at least three, each hyperedge has a unique logical label.

Lastly, we note that a decoding algorithm can choose to simplify the decoding hypergraph in various ways. One way that we always employ here is the process of deflagging. Flag measurements from qubits 16, 18, 21, 23 are simply ignored with no corrections applied. If flag 11 is non-trivial and 12 trivial, apply *Z* to 2. If 12 is non-trivial and 11 trivial, apply *Z* to qubit 6. If flag 13 is non-trivial and 14 trivial, apply *Z* to qubit 4. If 14 is non-trivial and 13 trivial, apply *Z* to qubit 8. See ref. ^15^ for details on why this is sufficient for fault-tolerance. This means that instead of including error-sensitive events from the flag qubit measurements directly, we preprocess the data by using the flag information to apply virtual Pauli *Z* corrections and adjust subsequent error-sensitive events accordingly. Hyperedges for the deflagged hypergraph can be found through stabilizer simulation incorporating the *Z* corrections. Let *r* indicate the number of rounds. After deflagging, the size of the set *V* for *Z* (resp. *X* basis) experiments are ∣*V*∣ = 6*r* + 2 (resp. 6*r* + 4), due to measuring six stabilizers per round and having two (resp. four) initial error-sensitive stabilizers after state preparation. The size of *E* is similarly ∣*E*∣ = 60*r* − 13 (resp. 60*r* − 1) for *r* > 0.

Considering *X* and *Z* errors separately, the problem of finding a minimum weight error correction for the surface code can be reduced to finding a minimum weight perfect matching in a graph^4^. Matching decoders continue to be studied because of their practicality^27^ and broad applicability^28,29^. In this section, we describe the matching decoder for our distance-3 heavy-hexagon code.

The decoding graphs, one for the *X*-errors (Fig. 1c) and one for the *Z*-errors (Fig. 1d), for minimum weight perfect matching are in fact subgraphs of the decoding hypergraph in the previous section. Let us focus here on the graph for correcting *X*-errors, since the *Z*-error graph is analogous. In this case, from the decoding hypergraph we keep nodes *V*_*Z*_ corresponding to (the difference of subsequent) *Z*-stabilizer measurements and edges (i.e. hyperedges with size two) between them. Additionally, a boundary vertex *b* is created, and size-one hyperedges of the form {*v*} with *v* ∈ *V*_*Z*_, are represented by including edges {*v*, *b*}. All edges in the *X*-error graph inherit probabilities and logical labels from their corresponding hyperedges (see Table 1 for *X* and *Z*-error edge data for 2-round experiment).

A perfect matching algorithm takes a graph with weighted edges and an even-sized set of highlighted nodes, and returns a set of edges in the graph that connects all highlighted nodes in pairs and has minimum total weight among all such edge sets. In our case, highlighted nodes are the non-trivial error-sensitive events (if there are an odd number, the boundary node is also highlighted), and edge weights are either chosen to all be one (uniform method) or set as ${w}_{e}=\log \left((1-{p}_{e})/{p}_{e}\right)$, where *p*_*e*_ is the edge probability (analytic method). The latter choice means that the total weight of an edge set is equal to the log-likelihood of that set, and minimum weight perfect matching tries to maximize this likelihood over the edges in the graph.

Given a minimum weight perfect matching, one can use the logical labels of the edges in the matching to decide on a correction to the logical state. Alternatively, the *X*-error (*Z*-error) graph for the matching decoder is such that each edge can be associated to a code qubit (or a meausurement error), such that including an edge in the matching implies an *X* (*Z*) correction should be applied to the corresponding qubit.

Maximum likelihood decoding (MLD) is an optimal, albeit non-scalable, method for decoding quantum error-correcting codes. In its original conception, MLD was applied to phenomenological noise models where errors occur only just before syndromes are measured^24,30^. This of course ignores the more realistic case where errors can propagate through the syndrome measurement circuitry. More recently, MLD has been extended to include circuit noise^23,31^. Here, we describe how MLD corrects circuit noise using the decoding hypergraph.

MLD deduces the most likely logical correction given an observation of the error-sensitive events. This is done by calculating the probability distribution Pr[*β*, *γ*], where $\beta \in {{\mathbb{Z}}}_{2}^{|V|}$ represents error-sensitive events and $\gamma \in {{\mathbb{Z}}}_{2}^{2k}$ represents a logical correction.

We can calculate Pr[*β*, *γ*] by including every hyperedge from the decoding hypergraph, Fig. 1c–f, starting from the zero-error distribution, i.e. Pr[0^∣*V*∣^, 0^2*k*^] = 1. If hyperedge *h* has probability *p*_*h*_ of occurring, independent of any other hyperedge, we include *h* by performing the update

6 $$\Pr [\beta,\gamma ]\leftarrow (1-{p}_{h})\Pr [\beta,\gamma ]+{p}_{h}\Pr [(\beta \oplus {\beta }_{h}),(\gamma \oplus {\gamma }_{h})],$$

where ${\beta }_{h}\in {{\mathbb{Z}}}_{2}^{|V|}$ is just a binary vector representation of the hyperedge. This update should be applied once for every hyperedge in *E*.

Once Pr[*β*, *γ*] is calculated, we can use it to deduce the best logical correction. If ${\beta }^{*}\in {{\mathbb{Z}}}_{2}^{|V|}$ is observed in a run of the experiment,

7 $${\gamma }^{*}={{{{{{{{\rm{argmax}}}}}}}}}_{\gamma }\Pr [{\beta }^{*},\gamma ]$$

indicates how measurements of the logical operators should be corrected. For more details on specific implementations of MLD, refer to Methods “Maximum likelihood implementations”.

### Experimental realization

For this demonstration we use ibm_peekskill v2.0. 0, a 27 qubit IBM Quantum Falcon processor^32^ whose coupling map enables a distance-3 heavy-hexagon code, see Fig. 1. The total time for qubit measurement and subsequent real-time conditional reset, for each round, takes 768ns and is the same for all qubits. All syndrome measurements and resets occur simultaneously for improved performance. A simple *X*_*π*_-*X*_*π*_ dynamical decoupling sequence is added to all code qubits during their respective idling periods.

Qubit leakage is a significant reason why the Pauli depolarizing error-model assumed by the decoder design might be inaccurate. In some cases, we can detect whether a qubit has leaked out of the computation subspace at the time it is measured (see Methods “Post-selection method” for more information on the post-selection method and limitations). Using this, we can post-select on runs of the experiment when leakage has not been detected, similar to ref. ^18^.

**Fig. 2:**
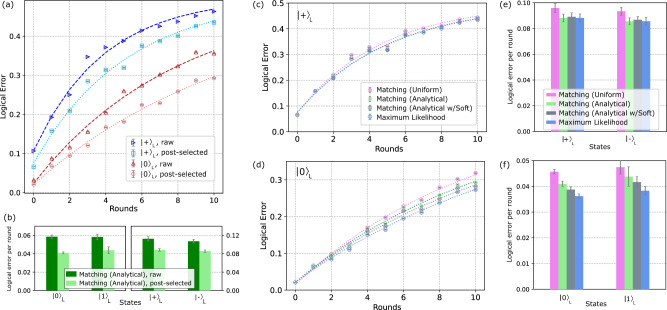
Logical error results. **a** Logical error versus number of syndrome measurement rounds *r*, where one round includes both a *Z* and an *X* stabilizer measurement. Blue right-pointing triangles (red triangles) mark logical errors obtained from using matching analytical decoding on raw experimental data for ${\left|\!+\!\right\rangle }_{L}$ (${\left|0\right\rangle }_{L}$) states. Light blue squares (light red circles) mark those for ${\left|\!+\!\right\rangle }_{L}$ (${\left|0\right\rangle }_{L}$) with the same decoding method but using leakage-post-selected experimental data. Error bars denote sampling error of each run (500,000 shots for raw data, variable number of shots for post-selected). Dashed line fits of error yield error per round plotted in **b**. **b** Applying the same decoding method on leakage-post-selected data, shows substantial reduction in overall error for all four logical states. See Methods “Post-selection method” for details on post-selection. Fitted rejection rate per round for ${\left|0\right\rangle }_{L}$, ${\left|1\right\rangle }_{L}$, ${\left|\!+\!\right\rangle }_{L}$, ${\left|-\right\rangle }_{L}$ are 4.91%, 4.64%, 4.37%, and 4.89%, respectively. Error bars denote one standard deviation on the fitted rate. **c**, **d** Using post-selected data, we compare logical error obtained with the four decoders: matching uniform (pink circles), matching analytical (green circles), matching analytical with soft information (gray circles), and maximum likelihood (blue circles). (See Fig. 6 for ${\left|-\right\rangle }_{L}$ and ${\left|1\right\rangle }_{L}$). Dashed fitted rates presented in **e**, **f**. Error bars denote sampling error. **e**, **f** Comparison of fitted error per round for all four logical states using matching uniform (pink), matching analytical (green), matching analytical with soft information (gray), and maximum likelihood (blue) decoders on leakage-post-selected data. Error bars represent one standard deviation on the fitted rate.

In Fig. 2a, we initialize the logical state ${\left|0\right\rangle }_{L}$ (${\left|\!+\!\right\rangle }_{L}$), and apply *r* syndrome measurement rounds, where one round includes both *X* and *Z* stabilizers (total time of approximately 5.3*μ*s per round, Fig. 1b). Using analytical perfect matching decoding on the full data set (500,000 shots per run), we extract the logical errors in Fig. 2a, red (blue) triangles. Details of optimized parameters used in analytical perfect matching decoding can be found in Methods “IBM_Peekskill and experimental details”. Fitting the full decay curves (eq. (14)) up to 10 rounds, we extract logical error per round without post-selection in Fig. 2b of 0.059(2) (0.058(3)) for ${\left|0\right\rangle }_{L}$ (${\left|1\right\rangle }_{L}$) and 0.113(5) (0.107(4)) for ${\left|\!+\!\right\rangle }_{L}$ (${\left|-\right\rangle }_{L}$).

Applying the same decoding method on leakage-post-selected data reduces logical errors in Fig. 2a, and leads to fitted error rates of 0.041(1) (0.044(4)) for ${\left|0\right\rangle }_{L}$ (${\left|1\right\rangle }_{L}$) and 0.088(3) (0.085(3)) for ${\left|\!+\!\right\rangle }_{L}$ (${\left|-\right\rangle }_{L}$) as shown in Fig. 2b. Rejection rates per round from post-selection for ${\left|0\right\rangle }_{L}$, ${\left|1\right\rangle }_{L}$, ${\left|\!+\!\right\rangle }_{L}$, and ${\left|-\right\rangle }_{L}$ are 4.91%, 4.64%, 4.37%, and 4.89%, respectively. See Methods “Post-selection method” for details.

In Fig. 2c–f, we compare the logical error for each round and extracted logical error per round obtained from the post-selected data sets using the three decoders described previously in Section “Decoding algorithms”. We also include a version of the analytical decoder that exploits soft-information^33^, which is described in Methods “Soft-information decoding”. We observe (see Fig. 2e, f) a consistent improvement in decoding moving from matching uniform (pink), to matching analytical (green), to matching analytical with soft information, to maximum likelihood (grey), though this is much less significant for the *X*-basis logical states. A quantitative comparison between the three decoders for all four logical states at *r* = 2 rounds is provided in Methods “Logical error at *r* = 2 rounds”.

There are at least three reasons the *X*-basis states perform worse than the *Z*-basis. The first is the natural asymmetry in the circuits. The larger depth required for measuring *Z* stabilizers leads to more time where *Z* errors on data qubits can accumulate undetected. This is supported by simulations, like those in^1^, which uses a different decoder, and here in Methods “Simulation details”, which see worse performance of the *X*-basis for this *d* = 3 code. Second, choices made in decoding, particularly the deflagging step, can exacerbate the asymmetry by essentially converting measurement and reset errors into *Z* errors on the data qubits. This leads to a high effective *Z*-error rate that cannot be improved much, even by maximum likelihood decoding. In contrast, if we deflag only the first round of measurements, the logical error of the maximum likelihood decoder on the *r* = 2 round, ${\left|\!+\!\right\rangle }_{L}$ experiment decreases by around 2.8% to 18.02(7)%. Flagged decoding like this becomes time-consuming for larger round counts as adding flag nodes to the decoding hypergraph greatly increases its size. Finally, decoders are only as good as our model of the experimental noise. Non-depolarizing noise sources such as spectator *ZZ* errors, which we know are present, are not modeled by any of our decoders and will more adversely affect *X*-basis states. More accurate estimation and inclusion of such experimental noise and its implications for fault-tolerance is an important subject for further research.

## Discussion

The results presented in this work highlight the importance of the joint progress of quantum hardware, both in size and quality, and classical information processing, both concurrent with circuit execution and asynchronous to it, as described with the studied decoders. Our experiments incorporate mid-circuit measurements and conditional operations as part of a QEC protocol. These technical capabilities serve as foundational elements for further enhancement of the role of dynamic circuits in QEC, for example towards real-time correction and other feed-forward operations that will be critical for large-scale FT computations. We also show how experimental platforms for QEC of this size and capabilities can trigger new ideas towards more robust decoders. Our comparison between a perfect matching and a maximum likelihood decoder sets a promising starting point towards the understanding of the trade-off between decoder scalability versus performance in the presence of experimental noise. Better noise modeling and the techniques of pre-decoding errors^34, 35^ might improve the performance and run-time of these decoders.

All these key components will play a crucial role in larger distance codes, where the quality of the real-time operations (qubit conditional reset and leakage removal, teleportation protocols for logical gates, and decoding), along with device noise levels, will determine the performance of the code, potentially enabling the demonstration of logical error suppression with increased code distance.

## Methods

### Minimum weight perfect matching edge probabilities and implementation

**Fig. 3:**
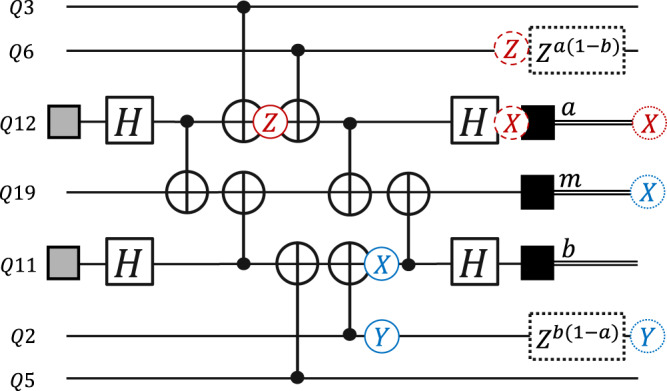
Example Pauli propagation. Two examples of Pauli propagation through the flagged measurement circuit for a *Z*-gauge operator. Pauli *Z* corrections due to deflagging are shown in dotted boxes and depend on the flag qubit measurement results. In the lower half of the figure in blue, a cx gate is followed by a *XY* error (blue) with probability *p*_cx_/15. The subsequent cx gate propagates the *X* error to the syndrome qubit *Q*19, flipping the measurement *m*, and meanwhile the *Y* error on *Q*2 propagates without change (it will have an effect on future measurement rounds). The propagated errors are in dotted circles. Note the flag measurement *b* is not flipped, as the Hadamard gate takes the *X* error to a harmless *Z* error. In the top half of the figure, a Pauli *Z* error occurs on a flag qubit (red) with probability *p*_cx_/15, and propagates to a *Z* error on *Q*6 and an *X* error before the measurement *a* (dashed circles). Deflagging applies *Z* to *Q*6, canceling the error there, so that the final propagated error is just the flip of measurement *a*.

We use the Gottesman-Knill theorem^25^ to propagate Pauli errors through our Clifford circuits and determine what error-sensitive events are made non-trivial. An example is shown in Fig. 3. If *p* is the probability of specific Pauli error and *e* is the corresponding set of non-trivial events, *p* is added to the edge probability *p*_*e*_.

Note that for experiments on states ${\left|0\right\rangle }_{L}$ and ${\left|1\right\rangle }_{L}$, we need only correct *X* errors and so just use the *Z* stabilizers, Fig. 1c. For experiments on ${\left|\!+\!\right\rangle }_{L}$ and ${\left|-\right\rangle }_{L}$, we need only correct *Z* errors with the graph in Fig. 1d. Edge probabilities are given for the ${\left|0\right\rangle }_{L}$ and ${\left |+\right\rangle }_{L}$ 2-round experiments in Table 1. We present just the edge weights for *r* = 2 rounds of syndrome extraction because this captures the behavior at time boundaries *t* = 1 and *t* = *r* + 1, as well as the behavior for 1 < *t* < *r* + 1. This latter bulk behavior is repeated over time for cases *r* > 2.

**Table 1:** Decoding graph edges

Edge *e*	*Q*(*e*)	First-order edge flip probability ${\tilde{p}}_{e}$	Num.
(${z}_{0}^{0}$, *b*)	1	44/15*p*_*cx*_ + 6*p*_*id*_ + 3*p*_*init*_ + 2*p*_*idm*_	0.039
(${z}_{0}^{0}$, ${z}_{1}^{0}$)	2	44/15*p*_*cx*_ + 14/3*p*_*id*_ + 3*p*_*init*_ + 2*p*_*idm*_	0.038
(${z}_{1}^{0}$, *b*)	3	44/15*p*_*cx*_ + 4*p*_*id*_ + 3*p*_*init*_ + 2*p*_*idm*_	0.037
(${z}_{0}^{0}$, ${z}_{0}^{1}$)	${{\emptyset}}$	88/15*p*_*cx*_ + 4/3*p*_*id*_ + 2*p*_*init*_ + 2*p*_*measure*_	0.061
(${z}_{1}^{0}$, ${z}_{0}^{1}$)	2	8/5*p*_*cx*_	0.016
(${z}_{1}^{0}$, ${z}_{1}^{1}$)	${{\emptyset}}$	88/15*p*_*cx*_ + 4/3*p*_*id*_ + 2*p*_*init*_ + 2*p*_*measure*_	0.061
(${z}_{0}^{1}$, *b*)	1	56/15*p*_*cx*_ + 28/3*p*_*id*_ + 4*p*_*idm*_	0.055
(${z}_{0}^{1}$, ${z}_{1}^{1}$)	2	56/15*p*_*cx*_ + 22/3*p*_*id*_ + 4*p*_*idm*_	0.053
(${z}_{1}^{1}$, *b*)	3	56/15*p*_*cx*_ + 28/3*p*_*id*_ + 4*p*_*idm*_	0.055
(${z}_{0}^{1}$, ${z}_{0}^{2}$)	${{\emptyset}}$	88/15*p*_*cx*_ + 4/3*p*_*id*_ + 2*p*_*measure*_ + 2*p*_*reset*_	0.061
(${z}_{1}^{1}$, ${z}_{0}^{2}$)	2	8/5*p*_*cx*_	0.016
(${z}_{1}^{1}$, ${z}_{1}^{2}$)	${{\emptyset}}$	88/15*p*_*cx*_ + 4/3*p*_*id*_ + 2*p*_*measure*_ + 2*p*_*reset*_	0.061
(${z}_{0}^{2}$, *b*)	1	44/15*p*_*cx*_ + 14/3*p*_*id*_ + 2*p*_*idm*_ + 3*p*_*measure*_	0.040
(${z}_{0}^{2}$, ${z}_{1}^{2}$)	2	44/15*p*_*cx*_ + 4*p*_*id*_ + 2*p*_*idm*_ + 3*p*_*measure*_	0.039
(${z}_{1}^{2}$, *b*)	3	44/15*p*_*cx*_ + 20/3*p*_*id*_ + 2*p*_*idm*_ + 3*p*_*measure*_	0.042
(${x}_{0}^{0}$, ${x}_{2}^{0}$)	4	4/3*p*_*h*_ + 8/5*p*_*cx*_ + 8/3*p*_*id*_ + *p*_*init*_ + 2/3*p*_*idm*_ + *p*_*measure*_	0.021
(${x}_{0}^{0}$, ${x}_{3}^{0}$)	5	2*p*_*h*_ + 12/5*p*_*cx*_ + 2*p*_*id*_ + 3*p*_*init*_ + 2/3*p*_*idm*_	0.028
(${x}_{0}^{0}$, *b*)	1	10/3*p*_*h*_ + 4*p*_*cx*_ + 16/3*p*_*id*_ + 4*p*_*init*_ + 4/3*p*_*idm*_ + *p*_*measure*_	0.049
(${x}_{0}^{0}$, ${x}_{0}^{1}$)	${{\emptyset}}$	8/3*p*_*h*_ + 16/15*p*_*cx*_ + *p*_*init*_ + 2*p*_*measure*_ + *p*_*reset*_	0.012
(${x}_{0}^{0}$, ${x}_{2}^{1}$)	4	8/15*p*_*cx*_	0.005
(${x}_{0}^{0}$, ${x}_{3}^{1}$)	5	8/15*p*_*cx*_	0.005
(${x}_{1}^{0}$, ${x}_{3}^{0}$)	6	4/3*p*_*h*_ + 16/15*p*_*cx*_ + 10/3*p*_*id*_ + *p*_*init*_ + 2/3*p*_*idm*_ + *p*_*measure*_	0.016
(${x}_{1}^{0}$, *b*)	3	4/3*p*_*h*_ + 8/5*p*_*cx*_ + 8/3*p*_*id*_ + 2*p*_*init*_ + 2/3*p*_*idm*_	0.020
(${x}_{1}^{0}$, ${x}_{1}^{1}$)	${{\emptyset}}$	4/3*p*_*h*_ + 8/15*p*_*cx*_ + *p*_*measure*_ + *p*_*reset*_	0.006
(${x}_{1}^{0}$, ${x}_{3}^{1}$)	6	8/15*p*_*cx*_	0.005
(${x}_{2}^{0}$, *b*)	7	4/3*p*_*h*_ + 16/15*p*_*cx*_ + 10/3*p*_*id*_ + 2*p*_*init*_ + 2/3*p*_*idm*_	0.016
(${x}_{2}^{0}$, ${x}_{2}^{1}$)	${{\emptyset}}$	4/3*p*_*h*_ + 8/15*p*_*cx*_ + *p*_*measure*_ + *p*_*reset*_	0.006
(${x}_{3}^{0}$, *b*)	8	10/3*p*_*h*_ + 52/15*p*_*cx*_ + 22/3*p*_*id*_ + 4*p*_*init*_ + 4/3*p*_*idm*_ + *p*_*measure*_	0.046
(${x}_{3}^{0}$, ${x}_{3}^{1}$)	${{\emptyset}}$	8/3*p*_*h*_ + 16/15*p*_*cx*_ + *p*_*init*_ + 2*p*_*measure*_ + *p*_*reset*_	0.012
(${x}_{0}^{1}$, ${x}_{2}^{1}$)	4	2/3*p*_*h*_ + 28/15*p*_*cx*_ + 8/3*p*_*id*_ + 4/3*p*_*idm*_ + *p*_*measure*_	0.025
(${x}_{0}^{1}$, ${x}_{3}^{1}$)	5	4/3*p*_*h*_ + 8/3*p*_*cx*_ + 2*p*_*id*_ + 4/3*p*_*idm*_ + 2*p*_*reset*_	0.032
(${x}_{0}^{1}$, *b*)	1	2*p*_*h*_ + 68/15*p*_*cx*_ + 20/3*p*_*id*_ + 8/3*p*_*idm*_ + *p*_*measure*_ + 2*p*_*reset*_	0.058
(${x}_{0}^{1}$, ${x}_{0}^{2}$)	${{\emptyset}}$	8/3*p*_*h*_ + 16/15*p*_*cx*_ + 2*p*_*measure*_ + 2*p*_*reset*_	0.012
(${x}_{0}^{1}$, ${x}_{2}^{2}$)	4	8/15*p*_*cx*_	0.005
(${x}_{0}^{1}$, ${x}_{3}^{2}$)	5	8/15*p*_*cx*_	0.005
(${x}_{1}^{1}$, ${x}_{3}^{1}$)	6	2/3*p*_*h*_ + 4/3*p*_*cx*_ + 10/3*p*_*id*_ + 4/3*p*_*idm*_ + *p*_*measure*_	0.020
(${x}_{1}^{1}$, *b*)	3	2/3*p*_*h*_ + 28/15*p*_*cx*_ + 10/3*p*_*id*_ + 4/3*p*_*idm*_ + *p*_*reset*_	0.025
(${x}_{1}^{1}$, ${x}_{1}^{2}$)	${{\emptyset}}$	4/3*p*_*h*_ + 8/15*p*_*cx*_ + *p*_*measure*_ + *p*_*reset*_	0.006
(${x}_{1}^{1}$, ${x}_{3}^{2}$)	6	8/15*p*_*cx*_	0.005
(${x}_{2}^{1}$, *b*)	7	2/3*p*_*h*_ + 4/3*p*_*cx*_ + 10/3*p*_*id*_ + 4/3*p*_*idm*_ + *p*_*reset*_	0.020
(${x}_{2}^{1}$, ${x}_{2}^{2}$)	${{\emptyset}}$	4/3*p*_*h*_ + 8/15*p*_*cx*_ + *p*_*measure*_ + *p*_*reset*_	0.006
(${x}_{3}^{1}$, *b*)	8	2*p*_*h*_ + 4*p*_*cx*_ + 22/3*p*_*id*_ + 8/3*p*_*idm*_ + *p*_*measure*_ + 2*p*_*reset*_	0.054
(${x}_{3}^{1}$, ${x}_{3}^{2}$)	${{\emptyset}}$	8/3*p*_*h*_ + 16/15*p*_*cx*_ + 2*p*_*measure*_ + 2*p*_*reset*_	0.012
(${x}_{0}^{2}$, ${x}_{2}^{2}$)	4	4/3*p*_*h*_ + 8/5*p*_*cx*_ + 8/3*p*_*id*_ + 2/3*p*_*idm*_ + 2*p*_*measure*_	0.021
(${x}_{0}^{2}$, ${x}_{3}^{2}$)	5	2*p*_*h*_ + 12/5*p*_*cx*_ + 2*p*_*id*_ + 2/3*p*_*idm*_ + *p*_*measure*_ + 2*p*_*reset*_	0.028
(${x}_{0}^{2}$, *b*)	1	10/3*p*_*h*_ + 4*p*_*cx*_ + 20/3*p*_*id*_ + 4/3*p*_*idm*_ + 3*p*_*measure*_ + 2*p*_*reset*_	0.052
(${x}_{1}^{2}$, ${x}_{3}^{2}$)	6	4/3*p*_*h*_ + 16/15*p*_*cx*_ + 10/3*p*_*id*_ + 2/3*p*_*idm*_ + 2*p*_*measure*_	0.017
(${x}_{1}^{2}$, *b*)	3	4/3*p*_*h*_ + 8/5*p*_*cx*_ + 10/3*p*_*id*_ + 2/3*p*_*idm*_ + *p*_*measure*_ + *p*_*reset*_	0.022
(${x}_{2}^{2}$, *b*)	7	4/3*p*_*h*_ + 16/15*p*_*cx*_ + 8/3*p*_*id*_ + 2/3*p*_*idm*_ + *p*_*measure*_ + *p*_*reset*_	0.016
(${x}_{3}^{2}$, *b*)	8	10/3*p*_*h*_ + 52/15*p*_*cx*_ + 6*p*_*id*_ + 4/3*p*_*idm*_ + 3*p*_*measure*_ + 2*p*_*reset*_	0.046

Edge data for the decoding graph in Fig. 1c, d correcting *X* (*Z*)-errors. Here ${z}_{s}^{t}$ (${x}_{s}^{t}$) indicates the *s*^th^*Z* (*X*)-stabilizer at time *t* as in Fig. 1c, d. If edge *e* is chosen by the matching decoder, a Pauli *X* (*Z*) is applied to qubit *Q*(*e*) if it is not ${{\emptyset}}$. Numeric values from the optimization in Section “IBM_Peekskill and experimental details” are provided in the last column.

To implement matching, we use PyMatching^28^ to perform the matching and decode. After the decoding graph is set up, decoding an entire leakage-postselected data set (i.e. typically somewhere between 100,000 and 200,000 unique bit strings) takes about 10 seconds, largely independent of *r* > 1.

### Maximum likelihood implementations

There are at least two different ways to implement maximum likelihood decoding (MLD), which we call the offline and online implementations of the decoder. Though they give the same results, the implementations can differ significantly in run-time depending on the specific application.

In the offline case, one calculates and stores the entire distribution Pr[*β*, *γ*] and queries it to determine the correction for each run of the circuit. The calculation takes *O*(∣*E*∣2^∣*V*∣+2*k*^) time, since we must perform updates from Eq. (6) to the distribution for each hyperedge in *E*. Determining a correction using Eq. (7) takes *O*(2^2*k*^) time per run.

Alternatively, one can forgo calculating the whole distribution, and instead calculate sparse distributions specific to each observation string β^*^ in a data set. Online MLD achieves this by pruning the distribution as updates are performed, keeping only entries consistent with β^*^. We imagine receiving one bit of β^*^ at a time. For the *j*^th^ bit, updates are made using Eq. (6) for all hyperedges that contain bit *j* and have not already been included. In fact, all these updates for a given bit can be combined into a pre-calculated transition matrix. Since no further updates will be made to bit *j*, we can now truncate the distribution by keeping only entries Pr[*β*, *γ*] where ${\beta }_{j}={\beta }_{j}^{*}$.

We can run through a quick example of this procedure for the 0-round, ${\left|0\right\rangle }_{L}$ experiment. Here there are just ∣*V*∣ = 2 error-sensitive events and ∣*E*∣ = 3 hyperedges. Organizing the hyperedge parameters like (*β*_*h*_, *γ*_*h*_): *p*_*h*_, we write

8 $$E=\{(10,\,1):{p}_{1},\,(11,\,0):{p}_{2},(01,\,0):{p}_{3}\},$$

where we have left out the *Z*_*L*_ bit of *γ*_*h*_ since *Z*_*L*_ corrections are not relevant to ${\left|0\right\rangle }_{L}$ experiments. This corresponds to just one round of the graph in Fig. 1c, and the expressions for *p*_1_, *p*_2_, *p*_3_ are the last three rows of Table 1. We will use $\bar{p}$ to mean 1 − *p* below.

Suppose we want to decode the observation β^*^ = 01. We start with probability distribution *P*_0_ = {(00, 0): 1}. This notation means Pr[*β* = 00, *γ* = 0] = 1. All other values of *β* and *γ* have probability zero and are not written. Perform updates according to hyperedges (10, 1) and (11, 0) to obtain

9 $${P}_{0} \mathop{\longrightarrow }\limits^{(10,1)} 	 \left\{(00,\,0)\right. :\left.{\bar{p}}_{1},\,(10,\,1):{p}_{1}\right\}\\ \mathop{\longrightarrow }\limits^{(11,0)} 	 \left\{(00,\,0)\right. :\left.{\bar{p}}_{1}{\bar{p}}_{2}\right),\,(10,\,1):{p}_{1}{\bar{p}}_{2},\\ 	 \; \, \; (11,\,0) :\left.{\bar{p}}_{1}{p}_{2},\,(01,\,1):{p}_{1}{p}_{2}\right\}.$$

Now we can truncate the distribution because we are done with all updates involving the first event. Since the first bit of β^*^ is 0, this leaves us with

10 $${P}_{1}=\{(00,\,0):{\bar{p}}_{1}{\bar{p}}_{2},\,(01,\,1):{p}_{1}{p}_{2}\}.$$

Now updates proceed for any other hyperedges involving the second event, just (01, 0) in this case.

11 $${P}_{1}\mathop{\longrightarrow }\limits^{(01,0)}\left\{(00,\,0):{\bar{p}}_{1}{\bar{p}}_{2}{\bar{p}}_{3},\,(01,1):{p}_{1}{p}_{2}{\bar{p}}_{3}\right.,$$

12 $$\left.\;\;\;\;\;\;\;\;\;\;\;\;\;\;\;\;\;(01,\,0):{\bar{p}}_{1}{\bar{p}}_{2}{p}_{3}:(00,\,1):{p}_{1}{p}_{2}{p}_{3}\right\},$$

which similarly is truncated to

13 $${P}_{2}=\{(01,\,1):{p}_{1}{p}_{2}{\bar{p}}_{3},\,(01,\,0):{\bar{p}}_{1}{\bar{p}}_{2}{p}_{3}\}.$$

To determine whether β^*^ requires a logical correction or not, compare ${p}_{{{{{{{{\rm{error}}}}}}}}}={p}_{1}{p}_{2}{\bar{p}}_{3}$ with ${p}_{{{{{{{{\rm{noerror}}}}}}}}}={\bar{p}}_{1}{\bar{p}}_{2}{p}_{3}$. As *p*_error_ is second order in experimental error rates and *p*_noerror_ is first order, we deduce that it is more likely that no logical error has occurred and apply no correction.

Suppose the number of nonzero entries in the probability distribution after truncating after the *j*^th^ bit is *S*_*j*_. During the course of online MLD, there is some maximum instantaneous size of the probability distribution, say ${S}_{\max }=\mathop{\max }\limits_{j}{S}_{j}$. The total time to determine a correction is $O(|V|{S}_{\max })$ per run, assuming a constant number of hyperedge updates per bit. Note that ${S}_{\max }$ depends on the decoding hypergraph and also the order in which error-sensitive events are incorporated. It can be argued that for [[*n*, *k*]] codes, repeated rounds of syndrome measurements, and events incorporated chronologically, ${2}^{n-k}\le {S}_{\max }\le {2}^{2n}$. The lower bound holds because after completing update and truncation for a complete round, any of the entire next round of *n* − *k* stabilizer bits may be flipped due to syndrome measurement errors. The upper bound follows from hyperedges being bounded to contain events from at most two consecutive rounds.

The online decoder is also amenable to dynamic programming, storing partially calculated probability distributions up to some moderately-sized *j*. This saves time by avoiding repeating the same calculations when observations with same prefixes are decoded. For instance, in the example above, we could store *P*_1_ since both observations β^*^ = 00 and 01 would end up calculating it. In our analysis of three-round experiments, we store distributions up to *j* = 15, while for four rounds we keep up to *j* = 21, in what is largely an attempt to balance time and memory consumption.

Since online MLD takes exponential (in *n*, the number of physical qubits in the code) time per run, if ∣*V*∣ is small enough, offline MLD is preferable. If ∣*V*∣ is large but *n* and *k* are small (perhaps a small code experiment performing many rounds of syndrome measurements), the online decoder becomes the only feasible option.

In the experiments here, online MLD becomes preferable over offline MLD for three rounds and greater. For *r* = 2, either offline or online MLD can decode a complete data set in around 90 seconds for logical *Z*-eigenstates (about 13,000 unique bit strings) and around 12 minutes for logical *X*-eigenstates (about 21,000 unique bit strings). However for *r* = 10, online MLD can take up to 3 weeks for a complete data set (around 130,000 unique bit strings).

All *r* ≥ 3 online MLD computations were run on a shared x86_64 Linux server. Using specialized hardware, like FPGAs is not an avenue we explored. However, given the ${S}_{\max }\ge {2}^{n-k}$ factor in the time complexity, we do not expect online MLD to be feasible for use in larger quantum devices.

### Simulation details

We obtain theoretical simulation results using stabilizer simulations of the Qiskit software stack^36^. In order to estimate the performance of quantum error correction circuits on IBM Quantum Falcon systems, we performed simulations of the quantum circuits with qubits mapped onto the Falcon devices using customized error models to reflect the noise behavior of experimental hardware.

Circuit errors in our simulation are modeled as depolarizing errors, so that the effect for different error sources of varying strength can be captured. Noise models were built following error locations and error channels described in Section “Decoding algorithms” using

- a depolarizing error model for each single and two qubit operation in the quantum circuit with error rates obtained from simultaneous randomized benchmarking (RB)
- a bit-flip error model for error in measurement, initialize, and reset operations
- a depolarizing noise model for idling error

Using the above described error model, we define a realistic depolarizing error model where simulations are carried out with noise parameters directly exported from the IBM Quantum processor used for this work, ibm_peekskill (Tables 2 and 3), including

- specific error rates for each single and two-qubit quantum operation with depolarizing quantum channel parameter obtained from simultaneous RB according to the relation
  $${\epsilon }_{{{{{{{{\rm{gate}}}}}}}}}=\frac{{2}^{n}-1}{{2}^{n}}(1-{\alpha }_{{{{{{{{\rm{gate}}}}}}}}}),$$
  where *ϵ*_gate_, *n*, *α*_gate_ represents error per gate, number of qubits in gate, and depolarizing quantum channel parameter,
- initialization, measurement, and reset error obtained as described in Table 2,
- idling errors with noise strength proportional to coherence limit of the gate, where coherence limit is computed using *T*_1_, *T*_2_ and idle time of each qubit during the execution of each quantum operation in the circuit. And each gate length matches that of the actual device (the circuit schedule matches that of experiment).

**Table 2:** Single qubit characterization

Qubit	Freq.	Anharm.	*T*_1_	*T*_2_	EPG	EPG simul	Readout	Initialization	Reset	${P}_{leak}^{m=10}$
(*Q*_*F*_)	(GHz)	(MHz)	(*μ*s)	(*μ*s)	(%)	(%)	error (%)	error (%)	error (%)	
1	4.664	−351.7	420.3	118.4	0.0102	0.0143	1.22	2.37	3.3	0.0884
2	4.799	−346.9	354.8	119.8	0.0128	0.0171	1.25	1.02	5.6	0.0203
3	4.862	−347.9	331.7	25.8	0.0096	0.0096^*^	0.75	1.27	5.4	0.0097
4	4.933	−345.9	124.8	77.3	0.0332	0.0315	0.47	0.52	2.0	0.0048
5	5.020	−343.9	131.7	215.5	0.0122	0.0145	0.79	1.23	1.2	0.0201
7	4.769	−347.1	424.5	59.7	0.0107	0.0212	0.47	0.22	3.3	0.1046
8	4.941	−344.3	249.4	228.8	0.0181	0.0310	0.46	0.67	1.1	0.0164
9	5.219	−339.4	271.7	316.0	0.0069	0.0287	1.28	1.89	1.7	0.0490
10	4.863	−347.1	357.0	72.0	0.0184	0.0207	0.30	0.53	1.8	0.0106
11	5.128	−341.4	283.8	188.8	0.0199	0.0217	1.38	2.82	2.6	0.0136
12	4.933	−344.8	280.9	353.0	0.0190	0.0367	0.38	0.26	1.2	0.0182
13	5.006	−356.5	349.8	345.0	0.0168	0.0410	0.10	0.74	0.9	0.0898
14	4.839	−377.2	399.3	99.7	0.0157	0.0694	1.22	3.67	4.6	0.0277
15	4.991	−368.8	226.6	217.4	0.0352	0.0473	0.37	1.48	3.2	0.0172
16	5.107	−342.0	259.8	209.2	0.0100	0.0280	0.70	0.88	1.2	0.0200
17	5.173	−339.3	234.4	311.7	0.0207	0.0324	0.65	0.71	1.6	0.0204
18	5.103	−339.9	195.5	34.7	0.0138	0.0118	0.26	0.40	1.2	0.0220
19	4.819	−376.7	319.6	167.6	0.0311	0.0485	2.15	8.87	7.2	0.0128
21	4.890	−345.8	278.1	308.0	0.0131	0.0143	0.53	0.77	0.7	0.0086
22	4.955	−344.2	206.9	132.4	0.0105	0.0177	0.62	1.30	1.8	0.0434
23	5.045	−341.8	278.3	145.0	0.0118	0.0118^*^	0.28	0.34	0.8	0.0192
24	5.136	−341.1	258.7	14.6	0.0147	0.0169	0.36	0.28	2.6	0.0096
25	5.027	−341.7	364.1	327.5	0.0160	0.0265	0.42	0.45	0.8	0.0164
Mean	4.966	−348.50	287.0	177.7	0.0165	0.0266	0.713	1.42	2.43	0.0288
Std.	0.139	10.8	79.9	107.0	0.0074	0.0140	0.48	1.81	1.73	0.0273

**Fig. 4:**
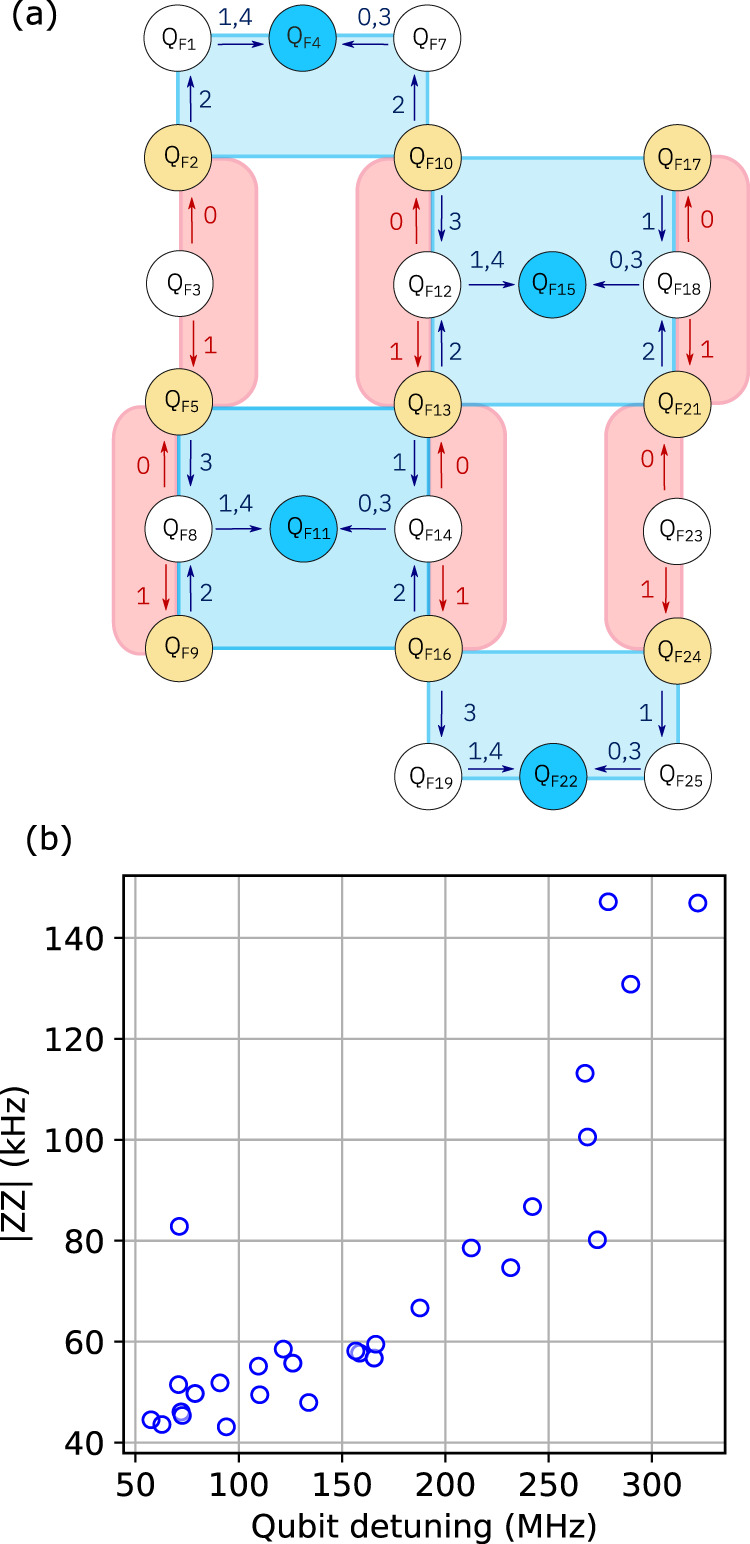
Experiment details. **a** Translation of Fig. 1a qubit numbering (*Q*_*N*_) to standard IBM-Falcon numbering(*Q*_*FN*_). **b** Static ZZ between all connected qubits pairs versus detuning between qubits. Median qubit anharmonicity, see Table 2 for breakdown, is -345 MHz.

**Fig. 5:**
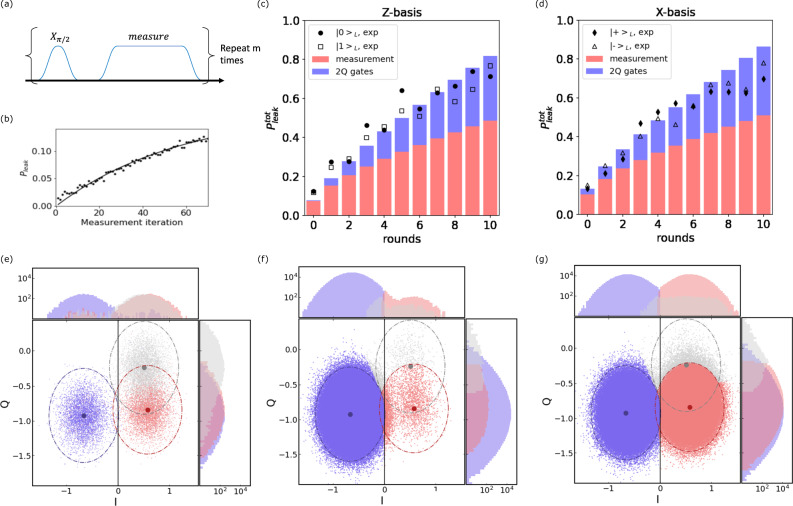
Leakage analysis. **a** Repeated measurement sequence for extracting leakage error during the measurement. The *X*_*π*/2_ pulse allows us to randomly sample leakage events from $\left|0\right\rangle$ or $\left|1\right\rangle$ states. **b** The leakage probability (${p}_{{{{{{{{\rm{leak}}}}}}}}}^{{{{{{{{\rm{meas}}}}}}}}}$) to the $\left|2\right\rangle$ state measured at *Q*_*F*14_. The leakage and seepage rate is obtained by fitting the data with Eq. (15). **c**, **d** Qubit leakage in the system as a function of syndrome measurement rounds for *Z* − and *X* − basis logical states. Bar plots show the ${p}_{{{{{{{{\rm{leak}}}}}}}}}^{{{{{{{{\rm{tot}}}}}}}}}$ as computed from the gate and measurement leakage rates, obtained from randomized benchmarking (2Q gates) and from the sequence shown in **a**, respectively. Experimental results, ${p}_{{{{{{{{\rm{leak}}}}}}}}}^{\exp }=1-{p}_{{{{{{{{\rm{accept}}}}}}}}}$, where *p*_accept_ is the acceptance probability calculated from the method outlined in Methods “Post-selection method”, are shown as black symbols for comparison. The experimental results plotted here do not include initialization leakage. **e** Readout calibration data for *Q*_*F*12_ (see Fig. 4a). The qubit is prepared in its $\left|0\right\rangle$, $\left|1\right\rangle$, and $\left|2\right\rangle$ states and measured. The collected statistics can be seen in as blue ($\left|0\right\rangle$), red ($\left|1\right\rangle$), and grey ($\left|2\right\rangle$) where the dot-dashed lines represent 3-*σ* for each distribution. **f** 3-state classification results for *Q*_*F*12_ after qubit initialization, and **g** after the first *X* − syndrome measurement.

Single qubit device parameters, using IBM-Falcon qubit numbering presented in Fig. 4a for ibm_peekskill. Single qubit error per gate (EPG) from randomized benchmarking (RB) with all coupled qubits idled. In contrast, simultaneous single qubit EPG (EPG simul) is obtained by performing one qubit RB concurrently with two qubit RB on neighboring gates to more realistically approximate simultaneous application of gates in stabilizers (asterisked values are isolated EPG, as these qubits are not captured in this scheme). To separate readout from initialization and reset errors, readout error is extracted from overlap of gaussian fits to ground and excited state histograms. The initialization sequence for the data presented in this paper used 6 rounds of conditional reset, with a single *Xπ*_*e*−*f*_ after 3 rounds to help reset f-state population. The initialization error is benchmarked with this sequence applied simultaneously on all qubits. Reset error is the average non-zero state population after a single round of conditional reset (simultaneous on all qubits) after preparing all qubits on with an *X*_*π*/2_, to capture mid-circuit reset needed for each stabilizer round. Probability of leakage after 10 measurements, see Eq. (15), is from benchmarking pulse-sequence in Fig. 5a.

Furthermore, to demonstrate average performance of the circuit in a relatively uniform depolarizing error model, we define an average depolarizing error model where instead of the specific error rates for different gates and qubits stated above we use average error rates throughout the entire device to define the depolarizing error channels.

Using the analytical perfect matching decoder parameters *p*_*C*_ = [0.0126, 0.000266, 0.0, 0.001, 0.002, 0.000266, 0.000266, 0.0, 0.00713, 0.0142, 0.0290] ordered by error locations $C=\{{{{{{{{\rm{cx}}}}}}}},\,{{{{{{{\rm{h}}}}}}}},\,{{{{{{{\rm{s}}}}}}}},\,{{{{{{{\rm{id}}}}}}}},\,{{{{{{{\rm{idm}}}}}}}},\,{{{{{{{\rm{x}}}}}}}},\,{{{{{{{\rm{y}}}}}}}},\,{{{{{{{\rm{z}}}}}}}},\,{{{{{{{\rm{measure}}}}}}}},\,{{{{{{{\rm{initialize}}}}}}}},\,{{{{{{{\rm{reset}}}}}}}}\}$ defined in Section “Decoding algorithms”, we obtained simulated per round logical error rates for circuits with up to 10 syndrome measurement rounds as 0.059 (0.038) for logical state ${\left|0\right\rangle }_{L}$ and 0.152 (0.106) for logical state ${\left|\!+\!\right\rangle }_{L}$ under the influence of realistic (average) depolarizing error model, respectively. Comparing to logical error per round on leakage post-selected data (with analytical matching decoding) as shown in Fig. 2a, of 0.0409 for ${\left|0\right\rangle }_{L}$ and 0.0882 for ${\left|\!+\!\right\rangle }_{L}$, logical error per rounds using the average depolarizing error model match the data better than the realistic model. However the average model still over estimates ${\left|\!+\!\right\rangle }_{L}$ state, showing that this simpler model does not fully capture the errors in the system. We believe the realistic model overestimates both Z and X-basis errors in part because the error benchmarks (see Tables 2 and 3) supplied to the model were themselves not leakage-aware and so the error benchmarks were likely inflated by leakage errors. We do see that the realistic model similarly does a better job predicting ${\left|0\right\rangle }_{L}$ than ${\left|\!+\!\right\rangle }_{L}$. This is an area of open work, for simulation and perhaps also decoding, to better capture the experimental data beyond leakage.

**Table 3:** Two qubit characterization

Gate	CX length (ns)	EPG (%)	EPG_simul (%)
11_14	483.6	0.53	0.95
12_15	433.8	0.78	1.42
12_10	334.2	0.42	1.02
12_13	519.1	0.61	1.03
14_13	504.9	0.77	1.49
15_18	469.3	0.49	1.10
16_14	440.9	0.50	0.82
16_19	696.9	2.09	1.14
18_17	426.7	4.03	3.96
18_21	348.4	0.56	0.73
2_1	362.7	0.34	0.55
21_23	519.1	0.65	0.67
22_19	362.7	0.50	0.94
22_25	412.4	0.47	0.57
24_23	384.0	0.64	0.81
24_25	384.0	0.69	0.91
3_2	426.7	0.52	0.53
3_5	391.1	0.40	0.64
4_1	547.6	0.46	0.49
5_8	348.4	0.47	0.71
7_10	362.7	1.42	0.76
7_4	426.7	0.39	0.54
8_11	526.2	1.16	1.39
9_8	384.0	0.58	0.84
Mean	437.3	0.81	1.00
Std.	82.5	0.77	0.68

The two qubit CX gates are constructed from the echoed cross-resonance gate^46^, with lengths and gate directions optimized for overall device performance. EPG is measured with spectator qubits idling while simultaneous EPG is taken with spectator qubits undergoing single qubit RB. Mean and standard deviation are across all CX gates listed.

### IBM_Peekskill and experimental details

Data in this section uses the qubit numbering (*Q*_*FN*_ contrasting with *Q*_*N*_ in Fig. 1) notation presented in Fig. 4a, matching standard IBM Quantum Falcon systems. Summarized in Table 2 are single qubit benchmarks for ibm_peekskill, where single qubit gates for all qubits (excluding virtual Z gates) are identically 35.55ns. While the Falcon layout has 27 qubits, for the d=3 circuits presented in this paper we only needed to use 23 of those qubits as shown in Fig. 4a, excluding qubits *Q*_*F*0_, *Q*_*F*6_, *Q*_*F*20_, and *Q*_*F*26_.

The always-on coupling between connected qubits on ibm_peekskill also results in undesirable static ZZ, plotted in Fig. 4b, as a function of qubit-qubit detuning. To mitigate some of these effects, a simple *X*_*π*_-*X*_*π*_ dynamical decoupling sequence is added to code qubits throughout the circuit. Furthermore, by introducing mixed dimensionality simultaneous RB^37^, we can further capture the undesired side-effects of this coupling by comparing one and two-qubit gate error taken with standard RB with spectator qubits/gates fully idling or with those simultaneously driven as set by scheduling requirements of the *Z* and *X* checks. Simultaneous gate error for gates and qubits not part of these measurements (always idling during the experiments presented in the main text) are thus not included in this extra characterization (in table as NaN). These results are presented in Tables 2 and 3. Optimization of two-qubit gates was undertaken on ibm_peekskill to ensure that no significant degradation in gate error or increase in leakage out of the computational manifold occurred in simultaneous benchmarking.

Using the same methodology presented in ref. ^15^, reset operations conditioned on the preceding measurement result are used for mid-circuit reset operations shown Fig. 1b. The total time of the measurement + reset cycle is 768ns, and includes an approximately 400ns measurement pulse, cavity ring-down time overlapping with classical control path delays, and application of the conditional *X*_*π*_. For consistency, all qubits are calibrated to use the same duration pulse and delays, with pulse amplitude calibrated individually to optimize QND-ness of readout.

To optimize the performance of the analytical perfect matching decoding on experimental data, an optimization algorithm was run on experimental data of one round stabilizer measurement of distance-3 heavy hexagon code on the same hardware to find a set of input error parameters that minimizes the decoder output logical error rates. Here we chose to use the L-BFGS-B algorithm^38^ due to efficiency of optimization and ability to work with simple linear constraints. This optimization was done by starting with the physical noise parameters found through device calibration, iteratively updating the parameters while minimizing the overall logical error. It aims to compensate for decoder’s lack of knowledge of realistic noise processes, and outputs a set of decoding parameters that produces improved decoder performance. The optimization resulted in the following set of input error parameters for the analytical perfect matching decoding algorithm *p*_*C*_ = [0.01, 0.00028, 0.0, 0.001, 0.002, 0.00028, 0.00028, 0.0, 0.0005, 0.0, 0.00001] following the error locations $C=\{{{{{{{{\rm{cx}}}}}}}},\,{{{{{{{\rm{h}}}}}}}},\,{{{{{{{\rm{s}}}}}}}},\,{{{{{{{\rm{id}}}}}}}},{{{{{{{\rm{idm}}}}}}}},\,{{{{{{{\rm{x}}}}}}}},\,{{{{{{{\rm{y}}}}}}}},\,{{{{{{{\rm{z}}}}}}}},\,{{{{{{{\rm{measure}}}}}}}},\,{{{{{{{\rm{initialize}}}}}}}},\,{{{{{{{\rm{reset}}}}}}}}\}$ as defined in Section “Decoding algorithms”.

We use the following equation to fit logical errors at syndrome measurement round, *r*,

14 $${P}_{{{{{{{{\rm{fail}}}}}}}}}(r)=\frac{1}{2}(1-A{e}^{-r/\tau })$$

where *A* is SPAM error, $\tau=\frac{-1}{ln(1-2\epsilon )}$, and *ϵ* is the logical error rate per syndrome measurement round (Fig. 2b, e, f).

### Leakage in the system

Leakage errors outside the computational space comprising the states $\left|0\right\rangle$ (*g*-state) and $\left|1\right\rangle$ (*e*-state) into $\left|2\right\rangle$ (*f*-state) or higher states cannot be corrected by our quantum error correction code and thus pose a serious threat to fault-tolerant computing. For fixed-frequency superconducting qubits, a certain set of qubit frequency assignments may lead to frequency collisions during the cross-resonant gate operation^2^. For example, when the target qubit frequency is close to the *e* → *f* transition frequency of the control qubit, leakage error is induced during the two qubit gate operation. Another example is a simultaneous operation of a two-qubit gate with a spectator single-qubit gate where the spectator qubit frequency together with target qubit frequency match the *e* → *f* transition of the control qubit. This can result in leakage errors which can be characterized by randomized benchmarking of the corresponding single- and two-qubit gates^39^.

Leakage errors can also occur during measurements^40^. As we speed up the measurement time by increasing the measurement power, qubits become more prone to leakage. We characterize this measurement-induced leakage by repeatedly measuring the qubit and extracting the leakage rate. The experiment is described in Fig. 5a, where the sequence consists of *X*_*π*/2_ followed by a measurement tone. The *X*_*π*/2_ pulse will map either $\left|0\right\rangle$ or $\left|1\right\rangle$ to the equator of the Bloch sphere, so the sequence randomly samples either $\left|0\right\rangle$ or $\left|1\right\rangle$ during the subsequent measurement. The obtained measurement leakage rate thus obtained is an average of the leakage rates from $\left|0\right\rangle$ and $\left|1\right\rangle$ states. The outcomes obtained from the sequence in Fig. 5a are classified according to calibration data obtained by preparing the $\left|0\right\rangle$, $\left|1\right\rangle$, and $\left|2\right\rangle$ states, using the closest distribution mean for each outcome, and then applying readout error mitigation by constraining the formalism described in ref. ^41^ for multi-qubit readout to our single-qubit three-state subspace. This single-qubit readout error mitigation is applied to the ensemble of measurements obtained for each iteration of the pulse sequence. The measurement sequence is repeated for *m* = 70 times and we average over the 10,000 shots for each *m* to compute the averaged probability that the qubit is binned in the $\left|2\right\rangle$ state. Figure 5b shows the measurement leakage probability, ${p}_{{{{{{{{\rm{leak}}}}}}}}}^{{{{{{{{\rm{meas}}}}}}}}}$, where the qubit leaks to the $\left|2\right\rangle$ state per measurement. (See Table 2 for further details). Eventually a steady state population in the $\left|2\right\rangle$ state, determined by the measurement leakage and seepage rates, is reached. We extract the leakage and seepage rates using the equation

15 $${p}_{{{{{{{{\rm{leak}}}}}}}}}^{{{{{{{{\rm{meas}}}}}}}}}=\frac{{{{\Gamma }}}_{L}}{{{{\Gamma }}}_{L}+{{{\Gamma }}}_{S}}\left(1-{e}^{-({{{\Gamma }}}_{L}+{{{\Gamma }}}_{S})m}\right),$$

where the leakage rate Γ_*L*_ is the probability of the qubit leaking during a measurement, the seepage rate Γ_*S*_ is the probability of a leaked state returning to the qubit subspace during a measurement. Here, Γ_*L*,*S*_ measures rate per measurement, therefore it is a unitless quantity. The obtained average and median value of Γ_*L*_ are 6.54 × 10^−3^ and 4.86 × 10^−3^ per measurement, respectively.

We extract the two-qubit gate leakage and seepage rate of the $\left|2\right\rangle$ state from simultaneous randomized benchmarking, with the simultaneity chosen to match the *Z* − and *X* − stabilizer sequences as illustrated in Fig. 1. Similarly, we extract the leakage/seepage rate from repeated measurement described in Fig. 5a. In these estimations, we account for the number of gate operations and measurements for each syndrome/flag qubits as well as the code qubits measured at the end. For instance, a two round experiment for the logical *Z* − basis consists of an *X* − check for state preparation, two rounds of *X* − and *Z* − checks, and a final measurement of the code qubits. Each check consists of two-qubit gates and measurements. As a result, there are three sets of two-qubit gates and measurements on *X* − check qubits, two sets of two-qubit gates and measurements on *Z* − check qubits, and one measurement of the code qubits. The post-selection procedure discards the result if any of the qubit is leaked from the computational subspace. Therefore, we sum all the leakage probabilities to compute ${p}_{{{{{{{{\rm{leak}}}}}}}}}^{{{{{{{{\rm{tot}}}}}}}}}$ for each syndrome measurement round.

Figure 5c, d shows ${p}_{{{{{{{{\rm{leak}}}}}}}}}^{{{{{{{{\rm{tot}}}}}}}}}$ as a function of the number of rounds for the logical *Z*- and *X*-basis, respectively. Black symbols denote leakage detected, as outlined in Methods “Post-selection method”, during the course of the error correction circuits themselves. This method onlys detect the occurrence of leakage and cannot differentiate the cause of leakage (measurement versus 2Q gate). With the analysis described in this section, we obtain estimates as shown by each bar in Fig. 5c, d, which represents ${p}_{{{{{{{{\rm{leak}}}}}}}}}^{{{{{{{{\rm{tot}}}}}}}}}$ from two-qubit gates (blue) and measurement (red) operations. When combined, the estimated leakage rate per round matches the experimental values decently well.

This analysis shows that reducing leakage error from both two-qubit gates and measurements is important. Decreasing leakage induced by two-qubit gates in our architecture will be associated with slower gates. With respect to measurement, as noted above, it is well known that a strong drive on a superconducting qubit system can lead to transitions both beyond the computational space^40^ and beyond the confinement of the Josephson cosine potential^42^. There is therefore a trade-off to be considered between readout error and measurement length and leakage probability. Slower readout impacts the system by increasing the idle time of the qubits not being measured. There have been proposals to deal with leakage in superconducting qubit systems by moving all the qubit excitations to the readout resonator, from which they decay to the environment^43^, or by designing readout resonator leakage reduction units (LRU)^44^ which exploit particular transition levels of the qubit-resonator system and which transform leakage errors into Pauli errors. LRU have also been proposed at the code level^45^. These options, as well as higher branching capabilities in readout and control electronics to conditionally reset qubits to the ground state from higher excitation levels, could be explored in experimental systems demonstrating quantum error correction in the near future.

### Post-selection method

We post-select all our results to remove detected leakage events in any of the qubits in our system. To do this, we look at 5000 integrated outputs for each qubit when prepared in each of the states $\left|0\right\rangle$, $\left|1\right\rangle$, and $\left|2\right\rangle$. We show this calibration for *Q*_*F*12_ (see Fig. 4a) in Fig. 5e. The overlap between the $\left|1\right\rangle$ and $\left|2\right\rangle$ states, which is significant in all 23 qubits used in this work, makes the classification of these states challenging. Furthermore, the presence of decay events ($\left|1\right\rangle$ to $\left|0\right\rangle$, $\left|2\right\rangle$ to $\left|1\right\rangle$, or $\left|2\right\rangle$ to $\left|0\right\rangle$) may impair the results using this training data within a supervised learning protocol. We instead apply clustering methods to our calibration data using a Gaussian Mixture Model (GMM) with three clusters, each cluster with an independent diagonal covariance matrix. The diagonal entries of the covariance matrices can be used to extract the standard deviations of the distribution for each qubit state. This offers a convenient way for us to define more flexible classification rules, compared to, for example, simpler clustering algorithms like K-means. Once the centroids and standard deviations (*σ*_*x*_ and *σ*_*y*_) are determined from the calibration data, we define regions for each state within the *I*/*Q* plane determined by a radius of 3*σ* on each axis around the corresponding centroid (see Fig. 5).

For any given measurement in any of the qubits, if the integrated outcome is within the $\left|0\right\rangle$-state region and the *I*-quadrature is negative, we classify that outcome as $\left|0\right\rangle$. If the integrated outcome is not within the $\left|0\right\rangle$-state region or the *I*-quadrature is positive, if it is within the $\left|1\right\rangle$-state region we classify it as $\left|1\right\rangle$, and if it is within the $\left|2\right\rangle$-state region but not within the $\left|1\right\rangle$-state region, we classify it as $\left|2\right\rangle$. For all other results, we classify the output according to its closest centroid.

This classification method is applied to every qubit after every measurement and the experimental runs in which any qubit is measured as $\left|2\right\rangle$ is discarded. Figure 5f shows the readout outcomes of *Q*_*F*12_ after the last initialization measurement. We only discard uncorrectable errors ($\left|2\right\rangle$ state) and retain experimental shots in which a qubit is in the $\left|1\right\rangle$ state after initialization, as that should be a correctable error by the code. Figure 5g shows the *Q*_*F*12_ results after the first *X* − check for a logical $\left|0\right\rangle$ state preparation. Both the initialization and the mid-circuit contain the 500,000 shots that are used for each error correction run in our experiments. For the initialization classification we obtain populations of 0.9910, 0.0071, and 0.0019 for the $\left|0\right\rangle$, $\left|1\right\rangle$, and $\left|2\right\rangle$ states, respectively. For the mid-circuit *X* − syndrome classification, those populations are observed to be 0.4972, 0.4962, and 0.0066.

### Logical error at *r* = 2 rounds

**Fig. 6:**
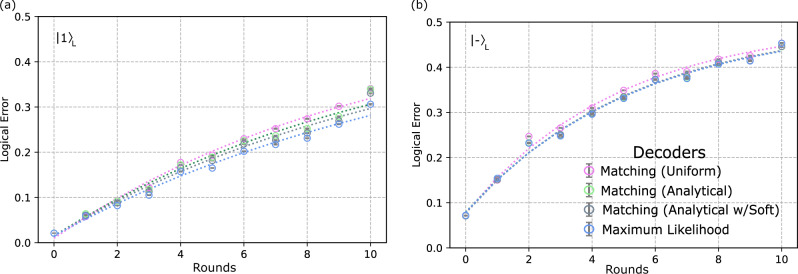
Logical error for ${\left|1\right\rangle }_{L}$ and ${\left|\!+\!\right\rangle }_{L}$ Comparison of logical error vs. round number for ${\left|1\right\rangle }_{L}$ and ${\left|-\right\rangle }_{L}$ states (${\left|0\right\rangle }_{L}$ and ${\left|\!+\!\right\rangle }_{L}$ in Fig. 2c, d) using four different decoding methods: matching uniform (pink), matching analytical (green), matching analytical with soft-decoding (gray), and maximum likelihood (blue). All decoders here are using leakage post-selected experimental data. Logical error per round extracted fits are shown in Fig. 2e, f.

Table 4 shows a comparison across the decoders studied in this work for state preparation and two rounds of syndrome measurement for the logical states ${\left|\!+\!\right\rangle }_{L}$, ${\left|-\right\rangle }_{L}$, ${\left|0\right\rangle }_{L}$, and ${\left|1\right\rangle }_{L}$. These results correspond to the values shown in Fig. 2c, d and Fig. 6 at *r* = 2 rounds.

**Table 4:** Decoder comparison for 2 round data

Basis	Init. State	Round Schedule	Matching Uniform (Full)	Matching Uniform (PS)	Matching Analytical (Full)	Matching Analytic (PS)	Maximum likelihood (Full)	Maximum likelihood (PS)	Shots (PS)
Z	${\left|0\right\rangle }_{L}$	XZXZX	0.1187(5)	0.0978(5)	0.1160(5)	0.0940(5)	0.1045(4)	0.0843(5)	322,165
Z	${\left|1\right\rangle }_{L}$	XZXZX	0.1151(5)	0.0928(5)	0.1162(5)	0.0920(5)	0.1031(4)	0.0819(5)	306,962
X	${\left|\!+\!\right\rangle }_{L}$	ZXZXZ	0.2555(6)	0.2212(7)	0.2502(6)	0.2091(7)	0.2502(6)	0.2083(7)	317,672
X	${\left|-\right\rangle }_{L}$	ZXZXZ	0.2860(6)	0.2468(8)	0.2805(6)	0.2332(8)	0.2803(6)	0.2321(8)	295,608

Comparison of logical error extracted using matching uniform, matching analytical, and maximum likelihood decoders on both full and leakage-post selected (PS) data-sets at *r* = 2 rounds. Error bars denote sampling error. Each full data set corresponding to 500,000 shots before post-selection, after post-selection each data point/set will have a different number of shots that is taken into account by the error bars.

### Soft-information decoding

In the main text, binary measurement outcomes (0 or 1) were deduced from experimental data and used in decoding. However, it has been shown^33^ that exploiting soft measurement information before it is converted into hard, binary information can improve decoding performance. Here we attempt this strategy with the matching decoder and find small improvements in our logical error rates per round.

Let us first describe how the soft information decoding works. For each measurement, three probabilities can be calculated based on the $\left|0\right\rangle,\left|1\right\rangle,\left|2\right\rangle$ classification Gaussians described in Section “Post-selection method”. These probabilities are $P[{{{\mathcal{M}}}}|i ]$, the probability of measurement ${{{{{{{\mathcal{M}}}}}}}}$ assuming the true qubit state was *i* = 0, 1, 2. After leakage post-selection, we assume the qubits were not in state $\left|2\right\rangle$, so only the *i* = 0, 1 probabilities feature into our modified matching algorithm.

We can use Bayes’ rule to write $P[i|{{{{{{{\mathcal{M}}}}}}}}]=P[{{{{{{{\mathcal{M}}}}}}}}|i]P[i]/P[{{{{{{{\mathcal{M}}}}}}}}]$, where *P*[*i*] and $P[{{{{{{{\mathcal{M}}}}}}}}]$ are a priori probabilities. We form the likelihood ratio^33^

16 $$L[{{{{{{{\mathcal{M}}}}}}}}]=\frac{P[1-h|{{{{{{{\mathcal{M}}}}}}}}]}{P[h|{{{{{{{\mathcal{M}}}}}}}}]}=\frac{P[{{{{{{{\mathcal{M}}}}}}}}|1-h]P[1-h]}{P[{{{{{{{\mathcal{M}}}}}}}}|h]P[h]},$$

where $h={{{{{{{{\rm{argmax}}}}}}}}}_{i\in \{0,1\}}P[{{{{{{{\mathcal{M}}}}}}}}|i]$ is the hard outcome corresponding to measurement ${{{{{{{\mathcal{M}}}}}}}}$. We also make the assumption that a priori *P*[0] = *P*[1]. This is not a very accurate assumption, especially for the flag qubits, which are expected to be $\left|0\right\rangle$ the majority of the time. However, this simplifies the likelihood ratio to $L[{{{{{{{\mathcal{M}}}}}}}}]=\frac{P[{{{{{{{\mathcal{M}}}}}}}}|1-h]}{P[{{{{{{{\mathcal{M}}}}}}}}|h]}$, a ratio of probabilities that are calculated directly from the experimental readout. Inputting more a priori information, perhaps expected probabilities from Pauli tracing, is a way to potentially improve soft information decoding.

We now modify the edge weights *w*_*e*_ and edge flip probabilities ${\tilde{p}}_{e}$ in the decoding graph (the same graph used by the uniform and analytical matching decoders). The first change is that ${p}_{{{{{{{{\rm{measure}}}}}}}}}$ in Table 1 is replaced by the appropriate likelihoods $L[{{{{{{{\mathcal{M}}}}}}}}]$. Note that while ${p}_{{{{{{{{\rm{measure}}}}}}}}}$ refers to the average probability a measurement fails, $L[{{{{{{{\mathcal{M}}}}}}}}]$ is different for each of the 18*r* + 15 (or 18*r* + 21) measurements ${{{{{{{\mathcal{M}}}}}}}}$ in a *Z*-basis (or *X*-basis) experiment, so Pauli tracing must be modified to assign unique likelihoods to each measurement. Finally, because we now use likelihoods, we replace all other terms *a*_*i*_*p*_*i*_ in ${\tilde{p}}_{e}$, for ${p}_{i}\,\ne \,{p}_{{{{{{{{\rm{measure}}}}}}}}}$, with *a*_*i*_*p*_*i*_/(1 − *p*_*i*_), and set ${w}_{e}=-\log {\tilde{p}}_{e}$.

Performing minimum-weight perfect matching with these modified edge weights on leakage post-selected data gives the logical error rates in Fig. 2c, d, and Fig. 6. We also attempted to use both hard ${p}_{{{{{{{{\rm{measure}}}}}}}}}/(1-{p}_{{{{{{{{\rm{measure}}}}}}}}})$ and soft $L[{{{{{{{\mathcal{M}}}}}}}}]$ likelihood terms in the edge probabilities, but this produced worse error rates. It’s possible the value of ${p}_{{{{{{{{\rm{measure}}}}}}}}}$ could be adjusted to improve this hard and soft combination decoding.

## Data Availability

Data available upon request to contributing authors.
